# Successful one stage operation for a synchronous, duodenal carcinoma, colonic carcinoma and renal oncocytoma in an adult patient

**DOI:** 10.1186/1477-7819-9-99

**Published:** 2011-09-01

**Authors:** Walid Faraj, Eman Sbaity, Deborah Mukherji, Ashraf Shamseddine, Ali Shamseddine, Mohamed Khalife

**Affiliations:** 1 American University of Beirut, Medical Centre Department of Surgery, HPB and Liver Transplant Unit, Beirut, Lebanon

**Keywords:** Colon cancer, duodenal cancer, oncocytoma, pancreaticoduodenectomy, synchronous tumors

## Abstract

We report a rare case of synchronous duodenal carcinoma, colonic carcinoma and renal oncocytoma successfully treated using a one-stage surgical approach. Potential risk factors for multiple primary malignancies associated with duodenal carcinoma are discussed. This case illustrates several practice points for consideration: 1. Patients presenting with small intestinal carcinomas have a higher than average chance of developing second primary tumors in other organs; this should be taken into consideration during staging and follow-up. 2. For full staging of patients presenting with small bowel tumors, upper and lower gastrointestinal endoscopy and PET scanning should be considered. 3. A one-stage surgical procedure can be used safely and successfully for multiple synchronous primary tumors.

## Background

Primary carcinomas of the duodenum, excluding carcinoma of the ampulla of Vater, have been reported to occur in 0.019-0.5% of all autopsies and in 35-45% of all cases of small intestinal cancer [1,2]. There have been few reported cases in the literature of multiple synchronous primary cancers of the duodenum and colon although a large population-based study has suggested that patients diagnosed with primary duodenal carcinoma have a higher than expected incidence of second primary malignancy [3]. We are reporting a case of synchronous duodenal and colonic carcinomas plus a renal oncocytoma successfully resected using a one-stage surgical approach.

## Case Report

**Figure 1:**
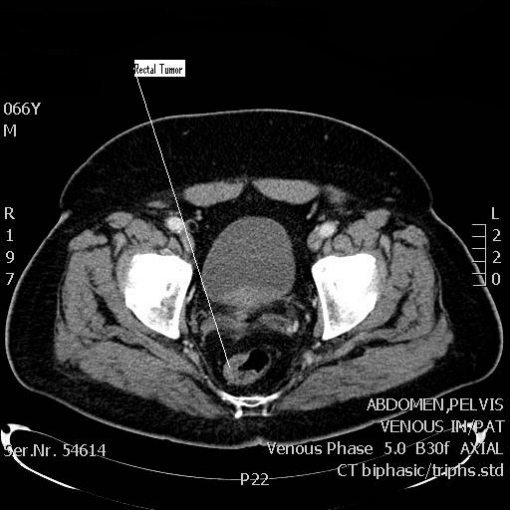
**CT scan showing the thickenned wall of the rectum suggesting the presence of rectal carcinoma**.

**Figure 2:**
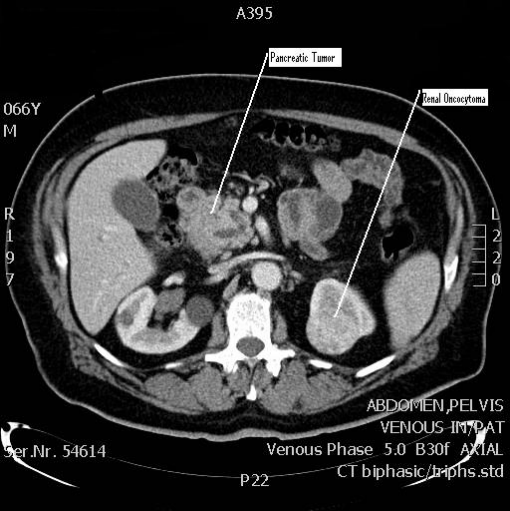
**CT scan of the abdomen showing the pancreatic tumor and the left renal oncocytoma**.

A 67 year old male presented with a history of weight loss and generalized weakness of 2 months duration. General investigations revealed anemia with hemoglobin of 9.2 g/dl. Upper gastrointestinal endoscopy was unremarkable, lower gastrointestinal endoscopy revealed a rectal polypoid mass (2.5 cm) with wide base, 12 cm from the anal verge (Figure 1). Biopsy of the mass revealed a moderately differentiated adenocarcinoma. Endoscopic rectal ultrasound confirmed the extension of the tumor to the muscularis propria and subserosa with no enlarged lymph nodes (T3N0). Staging computed tomography (CT) of chest, abdomen and pelvis showed a 4.7 × 3.6 cm mass in the pancreatic head with infiltration of the duodenum, and a left kidney mass (3 cm) suspicious of renal cell carcinoma (Figure 2). A positron emission tomography (PET) scan showed increased uptake in the pancreatic head mass (13 SUV) and in the rectal mass (12 SUV) with no uptake in the renal mass. The biochemical profile included the following: white blood count 7,700/μl, hemoglobin level 9.2 g/dl, protein of 35 g/dl, CEA = 1.17 ng/ml and CA 19-9 = 20 U/ml. A detailed family history was negative for malignancy.

The patient underwent a pancreaticoduodenectomy for the periampullary tumor, low anterior resection for the rectal tumour and partial nephrectomy for the renal tumour after an intraoperative frozen section revealed the presence of an oncocytoma.

The patient had the following reconstructive anastomosis: The pancreatic anastomotic reconstruction was via a loop of jejunum which was anastomosed to the pancreas in an end to side; duct to mucosa fashion, using 4/0 Polydioxanone (PDS) sutures. The biliary anastomosis was performed using 4/0 (PDS) sutures in an interrupted fashion end to side with the same jejunal loop. The gastro-jejunal anastomosis was performed in an end to side fashion using 3/0 PDS. The colonic anastomosis was performed with an EEA stapler. The operative time was 6 hours with minimal blood loss.

Pathology of the periampullary tumor revealed a moderately differentiated duodenal adenocarcinoma with 10 benign peri-pancreatic lymph nodes. Pathology of the colonic tumour showed a moderately differentiated infiltrating adenocarcinoma reaching but not crossing the muscularis propria with eight benign pericolonic lymph nodes (T2N0M0). Final pathology of the kidney mass was oncocytoma with had been completely excised.

The patient recovered well postoperatively and was discharged home.

## Discussion

Primary adenocarcinoma of the duodenum is very rare with an incidence of 0.035% of all gastrointestinal cancers [4]. It constitutes approximately 35-45% of small bowel cancer and presents in patients in their 5^th ^and 6^th ^decade with a median age of 55 years [5,6]. Multiple primary tumors of the duodenum and colon are also uncommon due to the rarity of duodenal cancer. In 1932, Warren and Gates set the criteria for multiple primary malignant tumors [7]. Presently, it is agreed on that each tumor must acquire specific features of malignancy, must be separate, and the possibilities that one tumor is a metastatic lesion deriving from another tumor must be excluded. Our case met these criteria; therefore we concluded that it is a case of multiple primary cancers.

The etiology and pathogenesis of small bowel and duodenal cancer is poorly understood. Several risk factors have been identified including Crohn's disease, familial adenomatous polyposis (FAP), celiac sprue, cystic fibrosis and colon cancer [8,9]. Several reports describe ampullary cancers as secondary primaries in patients with a history of colonic cancer in the setting of FAP [9,10]. Others describe secondary small bowel cancers with hereditary nonpolyposis colon cancer syndrome (HNPCC) [11]. Minniet al describes an increase incidence of small intestinal tumors; including duodenal adenocarcinoma, in patients with sporadic colonic malignancy [12].

Data from 13 cancer registries from Europe and Canada was analyzed in terms of incidence of second primary cancers following a diagnosis of small intestinal malignancy. This study reported a 68% overall increase in the risk of a new primary cancer after small intestinal carcinoma [3]. Increases were observed for cancers of the oropharynx, colon, and rectum, ampulla of Vater, pancreas, uterus, ovary, prostate, kidney, thyroid gland, skin and soft tissue sarcomas. The authors concluded that the apparent increase in risk may be partly attributable to overdiagnosis, genetic and environmental factors are likely to be important. The incidence of all cancers implicated in the HNPCC syndrome was increased after carcinoma of the small intestine and for colorectal, pancreatic and endometrial cancer the increased risk was mainly after early-onset small intestine cancer. The authors suggest that this supports the hypothesis that defects in mismatch repair and other DNA repair pathways, not necessarily leading to well characterized syndromes such as HNPCC, are common genetic features of cancers of the small intestine and other associated organs.

Dietary factors, alcohol consumption and high body mass index which are known risk factors for colon cancer are possibly acting as risk factors for small bowel adenocarcinoma in the same individual [3]. Renal oncocytoma is a benign epithelial tumor with excellent outcome. More than half of the patients are diagnosed incidentally. Those who present with symptoms usually present with abdominal pain, a palpable mass and gross hematuria. Nephron-sparing or partial nephrectomy is the accepted treatment for lesions less than 4 cm in diameter [13,14]. The pre-operative PET scan performed in this case showed the pancreatic head and rectal lesions to be equally FDG-avid however the renal lesion did not take up FDG. The sensitivity of PET for the detection of renal cell carcinoma has been debated however a recent study has shown a relatively high sensitivity and specificity compared to previous smaller reports [15]. In this case the lack of FDG uptake in the renal lesion demonstrated that it was not a renal metastasis from one of the others tumors; however a malignant renal lesion could not be excluded.

## Conclusions

In conclusion, we are presenting an unusual case report of a patient presenting with three synchronous primary tumors who treated with a successful on-stage surgical approach.

This case illustrates several practice points:

1. Patients presenting with small intestinal carcinomas have a higher than average chance of developing second primary tumors in other organs; this should be taken into consideration during staging and follow-up.

2. The use of upper and lower gastrointestinal endoscopy and consideration of PET scanning for full staging of patients presenting with small bowel tumors.

3. A one-stage surgical procedure can be successfully used for multiple synchronous primary tumors.

### Consent

Written informed consent was obtained from the patient for publication of this case report and accompanying images. A copy of the written consent is available for review by the Editor-in-Chief of this journal.

## Competing interests

The authors declare that they have no competing interests.

## Authors' contributions

ES drafted the manuscript, AcS and A1S participated in the design of the study, MK assisted with the collection of data and conceived of the study, WF and DM participated in the design and coordination of the study. All authors read and approved the final manuscript
